# PROMs Following Root Canal Treatment and Surgical Endodontic Treatment

**DOI:** 10.1016/j.identj.2022.06.015

**Published:** 2022-07-21

**Authors:** Jasmine Wong, Gary Shun Pan Cheung, Angeline Hui Cheng Lee, Colman McGrath, Prasanna Neelakantan

**Affiliations:** a Discipline of Endodontology, Division of Restorative Dental Sciences, Faculty of Dentistry, The University of Hong Kong, Hong Kong SAR, China; b Department of Dental Surgery, University of Hong Kong–Shenzhen Hospital, Shenzhen, China; c Dental Public Health, Faculty of Dentistry, The University of Hong Kong, Hong Kong SAR, China

**Keywords:** Minimal important difference, Oral health–related quality of life, Patient-centered outcome, Root canal treatment, Surgical endodontics

## Abstract

The FDI is currently working on developing a tool to encompass patient-reported outcome measures (PROMs) within the overall assessment of outcomes of endodontic treatment. The outcome of endodontic treatment has traditionally been determined by various clinical and radiographic criteria. However, these parameters do not address the impact of treatment on a patient's oral health–related quality of life (OHRQoL). OHRQoL, a crucial PROM, can be used to understand treatment outcome from a patient-centred perspective, thus improving clinician–patient communication whilst guiding decision-making. This focussed review aims to recount the OHRQoL of patients following nonsurgical root canal treatment and surgical endodontic treatment, with a specific focus on the minimal important difference (MID; the minimum score changes of an outcome instrument for a patient to register a clinically significant change in their OHRQoL and/or oral condition) and the methods used to determine it. The current evidence indicates that the OHRQoL of patients requiring root canal treatment is poorer than those without such need. Accordingly, the literature suggests that OHRQoL improves following nonsurgical or surgical endodontic treatment. However, study methodologies vary widely, and conclusions cannot be drawn with high confidence, nor can MID recommendations be provided. Well-designed clinical studies with baseline measurements and appropriate follow-up time frames are therefore needed. Despite that the literature is rife with outcome studies, research on PROMs is an area that deserves greater attention, particularly in relation to the MID. Determining the MID will facilitate the understanding of changes in outcome scores from the patients’ perspective, thus allowing for more informed decision-making in clinical practice.

## Introduction

Whilst the goal of root canal treatment is to eliminate infection, relieve pain, restore the health of the periapical tissues, and retain the functionality of the treated tooth,^1^^,^^2^ treatment effectiveness and success have traditionally been measured using clinician-reported outcomes that rely on clinical and radiographic criteria.^1^^,^^3^ Various criteria for successful root canal treatment have been proposed, most notably the “strict”^4^ and “loose”^5^^,^^6^ criteria, which are primarily categorised based on complete reversal of the periapical radiolucent area or its arrest. On the other hand, other terms such as favourable, uncertain, and unfavourable^1^ as well as healed, healing, nonhealed, and functional^7^ have also been proposed to describe endodontic treatment outcome. Dichotomisation of radiographic appearance as “success” or “failure” to convey prognosis may not be as relevant to patients, as they may have different goals, values, and/or treatment expectations than what the clinician may have in mind.^8^ Evaluating the treatment effectiveness from the patients’ perspective, that is, patient-centred outcomes, is of pivotal importance in the context of patient-centred care.^9^ Patient-centred care has been associated with improvements in patient satisfaction and overall well-being.^10^, ^11^, ^12^ In the context of endodontic therapy, patient-centred care emphasises the elimination of symptoms whilst prioritising functionality.^2^ A recent white paper by the FDI affirms that these treatment philosophies are in line with the concept of “endodontic medicine,” which suggests that endodontic diseases should be considered within a greater context, that is, the human body, as they not only affect the health of pulpal and periapical tissues but also impact general health.^2^

Quality of life (QoL) is one of the key components of patient-centred outcomes that form the basis of the patient–dentist dialogue.^13^ Whilst QoL indicators have been commonly employed throughout health care and general dentistry,^14^, ^15^, ^16^ it has only recently emerged as a topic of interest in endodontics. Current evidence indicates that root canal treatment would positively influence oral health–related QoL (OHRQoL).^17^, ^18^, ^19^ Despite such promising findings, a much-needed critical appraisal into the potential applications of OHRQoL and different OHRQoL instruments in the field of endodontic research is lacking. Most notably, the minimal important difference (MID) largely remains to be described in detail from the context of root canal treatments.^19^ The MID represents the smallest difference in a patient-reported outcome score that is considered clinically significant.^20^ Thus, to understand whether a change in OHRQoL is meaningful to the patient, determining the MID for the given context is essential. Currently there is a paucity in the evidence pertaining to the MID for OHRQoL measures, demonstrating the need for research in this area.^21^ Therefore, the aim of this review the current evidence to recount the OHRQoL of patients following nonsurgical root canal treatment and surgical endodontics, with a specific focus on MID.

## Review

### What is OHRQoL, why is it important, and how is it measured?

QoL is “an individual's perception of their position in life in the context of the culture and value systems in which they live and in relation to their goals, expectations, standard and concerns.”^22^ It is a multifaceted construct that incorporates physical, psychological, and social domains. It can be understood in terms of both positive—for example, having the ability to chew and function—and negative—for example, fatigue and pain—dimensions.^22^ Oral health can have an impact on overall health and QoL by impacting an individual's ability to carry out certain functions, such as chewing, talking, and tasting.^23^ Moreover, oral diseases can have psychological and social impacts that can affect an individual's well-being.^23^^,^^24^ Specifically, OHRQoL refers to an individual's self-perceived “comfort when eating, sleeping and engaging in social interaction, their self-esteem and their satisfaction with respect to their oral health.”^25^

Attempts to conceptualise the complex notion of OHRQoL to provide a reference framework for researchers and health care professionals have demonstrated that no one unique dimension can represent OHRQoL (ie, multidimensional) as the different domains work in tandem (ie, integrative).^14^^,^^26^^,^^27^ OHRQoL outcome measures are an essential component of patient-centred care since they allow clinicians to holistically evaluate the efficacy of different treatment options in light of the patient's needs and values.^13^ This improves patient–clinician communication and facilitates the treatment decision-making process.^28^ Furthermore, not all patients may have access to the “ideal” care because of social, cultural, and/or economic barriers; hence, patient-centred outcomes can facilitate the setting of individualised treatment goals.^28^ Where treatments may not be able to eliminate the disease but rather provide palliative/supportive care, improving the QoL may become the primary goal of treatment.^29^ In regards to public health, patient-centred outcomes may guide the development of health promotion programmes, allocate resources, and evaluate the efficacy of oral health care services.^13^ Furthermore, QoL indicators can be employed in dental research to facilitate evidence-based dentistry, cost-utility analysis, and health service evaluation.^30^

There are 3 main methods of evaluating OHRQoL: social indicators, global self-ratings, and multiple-item questionnaires.

a) Social indicators describe community-level social costs of oral disease. Population surveys are carried out to understand the social impact of diminished oral health such as loss of working days, restricted activities, and absence from schools.^31^ However, social indicators provide limited information about the impact of oral health on an individual's OHRQoL.^30^
b) Global assessment ratings (global self-ratings or single-item ratings) involve asking individuals one general question about their oral health status.^32^ They can be used to determine the responsiveness of an instrument as well as the MID of patient-centred outcomes.^33^ This method allows simple and general comparisons; however, it does not adequately reflect the various dimensions of OHRQoL. Therefore, global assessment ratings are often combined with multiple-item questionnaires.^34^
c) Multiple-item questionnaires represent the instrument of choice^31^ and can be categorised into generic- and disease- or condition-specific instruments, such as the Oral Health Impact Profile (OHIP)^35^ and Geriatric Oral Health Assessment Index,^36^ respectively.

### How has OHRQoL been measured in the endodontic literature?

A literature search was conducted to identify relevant studies using a search strategy that was developed based on previous reviews,^17^, ^18^, ^19^ utilising keywords that related to endodontic treatment and OHRQoL (Table 1). English publications investigating patient-reported outcome measures (PROMs) in relation to endodontic diseases and treatment were identified. After de-duplication and screening of the title and abstract, full texts of the relevant articles were obtained. The references of those articles were then hand searched for any other relevant studies. Hand-searched articles concerning the definition, concepts, and methods of measuring OHRQoL and MID were also included. The first search yielded a total of 449 studies. After removal of duplicates and hand searching of references, 32 clinical studies and 3 systematic reviews were identified. Tables 2 through 4 summarise the methodologic characteristics and key findings of the OHRQoL studies identified in the literature related to nonsurgical root canal treatment and surgical endodontics.

**Table 1:** Search strategy used to identify articles in this narrative review.

Search	Query
#1	“Root canal treatment” OR “root canal therapy” OR “endodontic treatment” OR “endodontics” OR “root canal retreatment” OR “Endodontic retreatment”
#2	“Apicoectomy” OR “apicectomy” OR “periradicular surgery” OR “Endodontic surgery” OR “apical surgery” OR “periapical surgery” OR “root-end surgery” OR “root-end resection”
#3	“Patient-reported outcome measures” OR “health-related quality of life” OR “oral health-related quality of life” OR “Quality of life” OR “quality of life index” OR “patient satisfaction” OR “general quality of life” OR “WHOQoL” OR “QoL” OR “health utility index” OR “SF-36” OR “SF-12” OR “SF-9” OR “SF-6” OR “EUROQoL” OR “EQ-5D”
#4	#1 OR #2
#5	#3 AND #4

**Table 2:** Summary of study characteristics involving nonsurgical root canal treatment and retreatment.

Study	Type of study; country	Sample	Intervention	Time frame	OHRQoL measure	MID determined	Key findings
Dugas et al 2002^37^	Cross-sectional study; Canada	119 patients	Root canal treatment	Treatment within 2 years from the beginning of the study	OHIP-17 on the impact of disease with corresponding questions on the impact of treatment	No	OHRQoL (OHIP-17 score) was most impacted by the disease in the domains of “physical pain” and “psychological disability.” Improvement after root canal treatment was experienced in all aspects of OHRQoL (OHIP-17 items). Patients with “painful aching” preoperatively had the largest rate of improvement. There were significant differences in the improvement of various aspects of OHRQoL associated with different factors (operator experience, PAI score, education, missing teeth).
Jordan et al 2009^54^	Prospective study; Republic of Gambia	15 patients	Root canal treatment: basic treatment protocol	Before treatment and 1 day, 5 days, 6 months, 12 months after treatment	HRQoL index	No	HRQoL improved (HRQoL index score decreased) with time after treatment, particularly in relation to pain, chewing ability and ability to work. The largest improvement was seen immediately after treatment (ie, 1 day). HRQoL index score fluctuated at 6 and 12 months.
Wright et al 2009^57^	Prospective study; USA	63 patients (15 endodontic, 16 denture, 32 recall) at baseline; 44 patients at follow-up	Root canal treatment, denture replacement, or recall with no apparent disease	Before treatment and 3 months after treatment	6- and 12- item OQOL instrument, global self-report of oral health	No	No significant difference of OHRQoL (OQOL scores) between groups at both time points. OHRQoL improved (OQOL instrument scores decreased) after treatment, with small effect sizes in the endodontic and recall group and moderate effect sizes in the denture group.
Gatten et al 2011^69^	Cross-sectional study; USA	37 patients (17 endodontic, 20 implant)	Root canal treatment vs implant treatment	After treatment, no specific time frame stated	OHIP-14, focus group discussions	No	The majority of patients did not experience any impact on OHRQoL (OHIP-14 score) after treatment. There was no significant difference of OHRQoL between groups. OHRQoL was most impacted in the domains of “physical pain” and “psychological disability” in both groups. The endodontic group experienced significantly higher scores in the domains “psychological discomfort” and “psychological disability” compared to the implant group. Most participants expressed a desire to retain their natural dentition when possible.
Yu et al 2012^55^	Cross-sectional study; Singapore	127 patients with 185 persistent lesions	Root canal treatment with persistent endodontic lesion and painful exacerbations	After treatment, no specific time frame stated	Modified OIDP	No	Out of the patients who experienced painful episodes, a large proportion reported no to very minor effect on their daily living.
Liu et al 2012^62^	Case-control study; Hong Kong	200 patients (100 endodontic patients, 100 control patients, ie, periodontal maintenance)	Indicated for root canal treatment vs periodontal maintenance	Before treatment or scheduled for periodontal maintenance	OHIP-14 Chinese version, GHQ-12 Chinese version	No	OHRQoL (OHIP-14 score) and psychological well-being (GHQ-12 score) were poorer in the endodontic patient group compared to the periodontal maintenance group.
Liu et al 2014^72^	Cross-sectional study; Hong Kong	412 patients	Indicated for root canal treatment	Before treatment	OHIP-14 Chinese version	No	OHRQoL (OHIP-14 scores) was poorer in endodontic patients and was associated with multiple teeth needing treatment, older age, and increased pain.
Liu et al 2014^40^	Prospective study; Hong Kong	253 patients at 1-month recall; 213 patients at 6-month recall	Root canal treatment	Before treatment and 1 month and 6 months after treatment	OHIP-14 Chinese version, global item rating of oral health improvement	No	OHRQoL significantly improved after treatment (OHIP-14 scores decreased) at both 1-month and 6-month recalls, with moderate and large effect sizes respectively. Self-ratings of improvement in oral health were significantly associated with changes in OHRQoL (OHIP-14 scores) and PAI scores.
Vena et al 2014^71^	Cross-sectional study; USA	1257 patients	Root canal treatment	Treatment within the last 3-5 years	OHIP-14	No	“Pain upon percussion” and “periapical pathosis” were associated with a negative impact on OHRQoL (OHIP-14 scores).
Montero et al 2015^39^	Prospective study (cross-sectional OHRQoL component); Spain	250 patients	Root canal treatment	Before treatment	OHIP-14_sev Spanish version	No	OHRQoL (OHIP-14_sev score) was most impacted at baseline in the domains of “physical pain” and “psychological discomfort.” There were significant differences in OHRQoL domains associated with various factors (tooth type, socioeconomic status, age, gender).
He et al 2017^67^	Prospective study; USA	52 patients	Root canal retreatment	At entry (before treatment) and 1 week, 1 month, 6 months, 12 months, and 24 months after treatment	OHIP-17	No	OHRQoL improved (OHIP-17 scores decreased) significantly after root canal retreatment. The largest improvement occurred within the first week, after which the improvement rate slowed.
Hamasha & Hatiwsh 2017^68^	Prospective study; Jordan	302 patients (101 were treated by 22 undergraduate students, 100 were treated by four graduate students and 101 participants were treated by three endodontic specialists)	Root canal treatment	Before treatment and 2 weeks after treatment	OHIP-17 in Arabic version	No	The median impact of pulpal disease on OHRQoL (OHIP-17 score) was low overall. The highest impact was observed in the domains “physical pain” and “psychological disability.” OHRQoL improved (OHIP-17 scores decreased) after treatment. No significant difference in improvement associated with operator level. There were significant differences in OHRQoL associated with various factors (presence of gingival inflammation, history of missing teeth, pulp status).
Chew et al 2019^66^	Prospective study; Australia	1096 patients at baseline, 438 at 2-year recall	Root canal treatment vs other dental services (extraction, restorations, prosthodontics, periodontics, preventative treatment, and scale and clean)	Baseline and 2 years	OHIP-14, global transition statement of change	No	Root canal treatment group had significantly lower odds for good/improved OHRQoL outcomes (lower OHIP-14 and GTSC scores) at the 2-year review compared to all dental services, but not individual treatment groups. The “preventative” and “scale and clean” groups had significantly higher odds for improved health.
Iqbal et al 2020^60^	Cross-sectional study; Pakistan	57 patients	Root canal treatment	After treatment, no specific time frame stated	OHRQoL research tool	No	A majority of patients expressed no impact on their OHRQoL after treatment in all 4 domains (physical function, psychological, social, and pain). A moderately good level of OHRQoL was observed amongst patients receiving root canal treatment. There were significant differences in the improvement of OHRQoL in the domains of physical function, psychological and pain associated with marital status, smoking status, and gender, respectively.
Wigsten et al 2020^63^	Prospective study: Sweden	85 patients (48 extraction, 37 endodontic)	Root canal treatment vs extraction	Baseline (at the initiation of treatment) and 1-month follow up	OHIP-14 and EQ-5D-5L Swedish versions	No	No significant difference of OHRQoL (OHIP-14 scores) between time points for both groups. The extraction group registered greater “embarrassment” compared to the endodontic group. HRQoL (EQ-5D-5L score) was significantly improved in the endodontic group only.

EQ-5D-5L, EuroQoL-5D-5L; GHQ-12, general health questionnaire-12; GTSC, global transition statement of change; HRQoL, health-related quality of life; MID, minimal important difference; OHIP-14, Oral Health Impact Profile-14; OHIP-17, Oral Health Impact Profile-17; OHIP-14_sev, Oral Health Impact Profile-14 severity; OIDP, Oral Impact on Daily Performance; OHRQoL, oral health–related quality of life; OQOL, Oral Health–Related Quality of Life instrument; PAI, periapical index.

**Table 3:** Summary of study characteristics involving procedural aspects of the nonsurgical root canal treatment protocol.

Study	Type of study; country	Sample size	Intervention	Time frame	OHRQoL measure	MID determined	Key findings
Pasqualini et al 2016^58^	Randomised clinical trial; Italy	47 patients (23 rotary, 24 reciprocating)	Reciprocating instrumentation vs rotary instrumentation	Every day from the day of surgery to 7 days postoperatively	POQoL questionnaire	No	OHRQoL improved with time (POQoL score decreased). Perceived OHRQoL was significantly better for the rotary group.
Bartols et al 2016^73^	Prospective study; Germany	137 patients (71 reciprocating group, 66 hand file group)	Reciprocating instrumentation vs hand instrumentation	In the week before treatment and in the week before completion of treatment (ie, 14 days after initial treatment)	OHIP-14 German version	No	OHRQoL improved (OHIP-14 scores decreased) significantly after root canal treatment. No significant difference of OHRQoL (OHIP-14 scores) between groups.
Yaylali et al 2017^61^	Randomised controlled trial; Turkey	70 (35 with foramen enlargement, 35 without)	Foraminal enlargement vs no foraminal enlargement	Every day from the day of surgery to 7 days postoperatively	QoLS	No	No significant difference of OHRQoL (QoLS scores) between groups.
Oliveira et al 2018^74^	Randomised clinical trial; Brazil	58 patients (29 reciprocating, 29 rotary)	Reciprocating instrumentation vs rotary instrumentation	24 hours after treatment	OHIP-14 Brazilian version	No	No significant difference of OHRQoL (OHIP-14 scores) between groups. Higher postoperative pain (VAS score) was associated with poorer OHRQoL (higher OHIP-14 score).
Yavari et al 2019^59^	Randomised clinical trial; Iran	196 patients (64 dexamethasone, 66 betamethasone, 64 saline)	Local infiltration of betamethasone vs dexamethasone vs saline after 1-visit root canal treatment	Before treatment; 6, 12, 24, 48, and 72 hours after treatment; and 7 days after treatment	POQoL questionnaire	No	Both the corticosteroid groups had significantly higher OHRQoL (POQoL scores) than the placebo group. A decrease in pain was associated with an increased in OHRQoL (POQoL scores).
Diniz-de-Figueiredo et al 2020^65^	Randomised controlled trial; Brazil	88 patients at 6 months (46 manual group, 42 reciprocating group); 87 patients at 12 months (42 manual group, 45 reciprocating group)	Reciprocating instrumentation and single cone obturation vs hand file instrumentation and lateral compaction obturation	Prior to treatment and 6 and 12 months after treatment	OHIP-14 Brazilian version	Yes	OHRQoL improved (OHIP-14 scores decreased) significantly after root canal treatment with moderate to large effect sizes. At 6 months, the manual protocol was associated with poorer OHIP-14 scores. At 12 months, there was no significant difference between groups. There was no significant difference between 6-month and 12-month OHIP-14 scores within groups.

MID, minimal important difference; OHIP-14, Oral Health Impact Profile-14; OHRQoL, oral health-related quality of life; POQoL, Postoperative Quality of Life; QoLS, Quality of Life Scale; VAS, visual analogue scale.

**Table 4:** Summary of study characteristics involving surgical endodontic treatment.

Study	Type of study; country	Sample size	Intervention	Time	OHRQoL measure	MID determined	Key findings
Tsesis et al 2005^53^	Prospective study; Israel	63 patients (31 traditional surgery, 32 microsurgery)	Surgical endodontic treatment with traditional vs microsurgical techniques	Every day from the day of surgery to 7 days postoperatively	PPQ	No	The traditional surgery group has significantly more pain than the microsurgery group. The microsurgery group had significantly more difficulty in mouth opening, speaking, and mastication than the traditional surgery group.
Del Fabbro et al 2009^48^	Randomised clinical trial; Italy	38 patients (19 PBI, 19 SI)	Surgical endodontic treatment with PBI vs SI flap designs	Every day from the day of surgery to 7 days postoperatively	PPQ	No	The PBI group experienced significantly faster reduction in pain compared, less swelling, and less chewing impairment than the SI group.
Del Fabbro et al 2012^47^	Randomised clinical trial; Italy	36 patients (18 control, 18 PRGF)	Surgical endodontic treatment with PRGF vs none	Every day from the day of surgery to 7 days postoperatively	PPQ	No	The PRGF group reported significantly less pain and swelling, less consumption of analgesics, and improved functional activities compared to the control group.
Taschieri et al 2014^51^	Retrospective study; Italy	20 patients (12 control, 8 PRGF)	Sinus perforation management with PRGF vs none during surgical endodontic treatment	Every day from the day of surgery to 7 days postoperatively	PPQ	No	The PRGF group reported significantly better OHRQoL in multiple domains (eg, swelling, bad breath/taste, pain, various functional activities) compared to the test group.
Meschi et al 2018^49^	Randomised controlled trial; Belgium	50 patients (25 LPRF, 25 control)	Surgical endodontic treatment with LPRF vs none	Every day from the day of surgery to 7 days postoperatively	PPQ	No	No significant difference of OHRQoL (patient perceived postoperative symptoms) between groups.
Metin et al 2018^56^	Prospective study; Turkey	71 (34 LLLT, 37 control)	Surgical endodontic treatment with LLLT vs none	1, 3, and 7 days postoperatively	GOHAI and OHIP-14 Turkish versions	No	The LLLT group reported significantly better OHRQoL (OHIP-14 and GOHAI scores) compared to the control on day 1 and 3 postoperatively.
Soto-Peñaloza et al 2020^50^	Randomised clinical trial; Spain	50 patients (25 A-PRF+, 25 control)	Surgical endodontic treatment with A-PRF+ vs none	Every day from the day of surgery to 7 days postoperatively	PPQ	No	The A-PRF+ group reported significantly better speech and sleep functions compared to the test group.
Khoo et al 2020^44^	Cross-sectional study; Singapore	150 patients (75 retreatment, 75 apical surgery)	Root canal retreatment vs surgical endodontic treatment	6 to 24 months after treatment	OHIP-14 Chinese and Malay versions	No	Impact on OHRQoL (OHIP-14 scores) was low, with no significant difference between groups. Impact most commonly experienced in the domains of “physical pain” and “psychological discomfort.” Poorer OHRQoL (higher OHIP-14 scores) associated with women and presence of preoperative pain. There was no correlation between OHIP-14 sores and healing outcome.
Tuk et al 2021^52^	Prospective study; Amsterdam	133 patients	Surgical endodontic treatment	Every day from the day of surgery to 7 days postoperatively	OHIP-14 Dutch version supplemented with questions on postoperative symptoms	No	OHRQoL generally improved throughout the week (OHIP-14 score decreased). There were significant differences in OHRQoL associated with various factors (age, postoperative infection, smoker).
Bharathi et al 2021^46^	Randomised clinical trial; India	40 patients (20 piezosurgery, 20 control)	Surgical endodontic treatment with piezosurgery protocol vs conventional	Every day from the day of surgery to 7 days postoperatively	PPQ	No	The piezosurgery group experienced significantly less swelling and less pain compared to the control group.

A-PRF+, advanced platelet-rich fibrin; GOHAI, General Oral Health Assessment Index; LLLT, low-level laser therapy; LPRF, leukocyte- and platelet-rich fibrin; MID, minimal important difference; OHIP-14, Oral Health Impact Profile-14; OHRQoL, oral health–related quality of life; PBI, papilla-based incision; PPQ, Patient Perception Questionnaire; PRGF, plasma rich in growth factors; SI, sulcular incision.

Dugas et al^37^ conducted the first study investigating the OHRQoL of endodontic patients and, since then, the importance of PROMs has been thrust into the limelight, prompting a steady growth of OHRQoL studies in relation to endodontic disease and treatment. The ideal instrument for PROMs should be appropriate, reliable, valid, responsive, and interpretable.^9^^,^^31^ However, as a “gold-standard” instrument for endodontic patients remains to be established, a myriad of measures has been employed to characterise the impact of root canal treatment on OHRQoL.^19^ Currently, the most frequently utilised instrument in the endodontic literature is the OHIP, specifically the OHIP-14.

The OHIP-14, which is a shortened version of the original OHIP-49, was developed based on Locker's conceptual model of oral health.^26^ The questionnaire is subdivided into 7 domains: functional limitation, physical pain, psychological discomfort, physical disability, social disability, and handicap. The patient answers based on how often they have encountered each scenario within a specific time frame, usually 12 months, using a 5-point Likert scale. The scores are summated, with a higher total score indicating poorer levels of OHRQoL.^38^ Other variations of the OHIP have been found in the endodontic literature as well, such as the OHIP-17^37^ and the OHIP-14_sev.^39^

A key benefit of using the OHIP-14 in the context of endodontics is that it has been confirmed to be sensitive enough to detect changes in patients’ OHRQoL following endodontic treatment.^40^ It also has been translated and validated in multiple languages,^41^, ^42^, ^43^ allowing adaptability for different cultural contexts. However, there exists much variation on how researchers interpreted the outcomes from OHIP-14. Some have dichotomised the results into “no impact” and “impact,”^44^ whilst others defined poor OHRQoL as scores that were amongst the upper quartile of the study group.^40^ Furthermore, how studies deduced improvement in OHQRoL was not standardised in the endodontic literature, with some inferring it from changes in the total score whilst others based it on changes to the individual domains or even the individual item level.^19^ It has been suggested that summed scores and domain-level analysis are favoured over item-level analysis.^45^

Other OHRQoL instruments have made also appeared in the endodontic literature, such as the Patient Perception Questionnaire,^46^, ^47^, ^48^, ^49^, ^50^, ^51^, ^52^, ^53^ Health-related QoL Index,^54^ Oral Impact on Daily Performance (OIDP),^55^ General Oral Health Assessment Index,^56^ OHRQoL instrument,^57^ Post-operative QoL questionnaire,^58^^,^^59^ OHRQoL research tool,^60^ and the QoL Scale.^61^ Although these instruments may provide an alternative means to measure PROMs, there are several factors that may hinder their widespread use in endodontic research. First, the responsiveness of most of these instruments have not been thoroughly investigated regarding OHRQoL changes associated with endodontic disease and treatment. Second, given their limited use throughout the endodontic literature, comparisons between studies may be challenging, which could prevent an accurate quantitative synthesis (ie, meta-analyses).

Health-related QoL measures, such as the General Health Questionnaire^62^ and the EuroQoL-5D-5L instrument,^63^ are sometimes utilised to provide an additional assessment of the patients’ general QoL. These instruments may allow researchers to evaluate how endodontic treatment–related factors can affect a patients’ self-perceived general health and overall well-being. However, the sensitivity of generic questionnaires is known to be inferior to disease-specific questionnaires.^64^

Apart from the choice of instrument, a crucial element for consideration is the time period of assessment. Ideally, a baseline measurement of the patients’ OHRQoL must be provided. Cross-sectional studies only capture the OHRQoL at a single time point, generally months to years posttreatment, which may result in susceptibility to recall bias. Prospective studies and randomised clinical trials thus possess a clear advantage. However, significant variation exists in regard to the evaluation periods. Whilst several studies have applied extended evaluation periods, for example, 1 year^54^^,^^65^ to 2 years,^66^^,^^67^ others reported postoperative assessments of only up to 7 days, sometimes without any preoperative baseline measurement.^46^, ^47^, ^48^, ^49^, ^50^, ^51^, ^52^ It has been suggested that limited time frames, for example, 6 months or less, are insufficient to evaluate changes in OHRQoL as they are limited to describing the initial posttreatment recovery. Hence, follow-up periods of approximately 1 year may be more suitable.^18^^,^^45^ On the other hand, further lengthening the period of evaluation may result OHRQoL fluctuations due to other oral diseases having emerged.^54^

### OHRQoL associated with endodontic disease and treatment

Endodontic diseases have been found to negatively impact OHRQoL^62^^,^^68^ particularly in the domains of physical pain, psychological disability, and psychological discomfort.^37^^,^^39^^,^^44^^,^^68^^,^^69^ Studies have reported OHRQoL improvement after primary^37^^,^^40^^,^^57^^,^^66^ and secondary^67^ nonsurgical root canal treatment as well as surgical endodontic treatment.^52^ Conversely, studies have also reported no significant difference in OHRQoL after nonsurgical treatment.^60^^,^^63^ These contrasting findings may be explained by the heterogeneity of endodontic patients in the disease- (ie, preoperative symptoms), treatment- (ie, complications), and patient-related factors (ie, experience of the treatment, psychosocial factors, and patient values). Furthermore, some endodontic diseases may manifest as “painless” ailments^70^ resulting in minimal perceived impact on OHRQoL.^44^^,^^68^ Therefore, it is likely that the extent of impact also depends on the severity of the symptoms, functional limitation, and psychosocial impairment. Nevertheless, based on the available literature, it may be considered that endodontic treatment generally improves the OHRQoL.^17^, ^18^, ^19^

When root canal treatment was compared with other dental services such as extraction, restoration, prosthodontic, periodontal, and preventative treatment, there were no differences when compared to individual treatment groups.^66^ Similarly, no significant difference was found in the OHRQoL between patients who had received root canal treatment vs extraction, although those in the extraction group expressed higher levels of embarrassment.^57^ It has also been reported that a consistent theme with most patients was the desire to keep their natural dentition.^69^

### Factors that may influence the OHRQoL of endodontic patients

A large cross-sectional study identified 3 key factors that were associated with poorer OHRQoL: multiple teeth needing treatment, retreatment, and pain.^62^ Both preoperative pain^44^^,^^62^ and persistent pain following treatment^55^^,^^71^ were found to negatively impact PROMs. The association between OHRQoL and different sociodemographic factors such as gender, age, socioeconomic status, and marital status and has been demonstrated in some studies^39^^,^^44^^,^^52^ and refuted in others.^68^^,^^72^ Similarly, studies on the impact of operator experience have reported conflicting findings, although patient satisfaction was consistently higher when treated by specialists.^37^^,^^68^

Multiple clinical studies have investigated how various procedural aspects of root canal treatment may impact OHRQoL. This includes local infiltration of corticosteroids,^59^ different instrumentation protocols,^58^^,^^65^^,^^73^^,^^74^ obturation techniques,^65^ and extent of foraminal enlargement.^61^ In terms of surgical endodontics, the use of microsurgical protocols,^53^ peizosurgery instruments,^46^ papilla-based flap designs,^48^ low-level laser therapy,^56^ and autologous platelet concentrates have also been evaluated in the context of PROMs. Except for the study by Diniz-de-Figueiredo et al,^65^ the period of evaluation was relatively short, spanning 2 weeks at most. Again, short evaluation times may be insufficient to thoroughly assess OHRQoL beyond patients’ initial recovery.^45^ Although it is entirely conceivable that different procedural aspects can impact the immediate postoperative experience of the patient, the influence of these factors on the long-term transformation of OHRQoL remains questionable.

A potential relationship between OHRQoL and clinical outcome has been implicated but not well substantiated in several studies.^37^^,^^40^ One study found an association between poorer OHRQoL and patients who had an endodontically treated tooth with persistent disease.^37^ The authors, however, encouraged caution in the interpretation of these results as radiographic outcomes given that this was a cross-sectional survey.^37^ Another study reported that all domains of OHIP-14 were significantly associated with self-perceived improvement in oral health, whilst some domains changed with respect to improvement in radiographic outcome.^40^ In general, clinical and radiographic parameters of success do not always reflect the changes in OHRQoL, whilst subjective measures such as self-perceived oral health appear to show a stronger correlation/association.^40^^,^^75^^,^^76^

### MID: a critical element for future research

The extent of benefit gained from any treatment is important for all stakeholders (eg, clinicians, patients, policymakers) to make changes in treatment philosophies. From the context of PROMs, the magnitude of change is a crucial element that represents the benefit gained from treatment. Statistical methods such as calculating the effect size and half of the standard deviation have also been utilised to indicate the magnitude of change.^40^^,^^57^^,^^65^ Global statements of change are widely used to assess the patients’ self-perceived change in oral health status.^40^^,^^57^^,^^66^ These methods can also be used to infer the responsiveness of the OHRQoL instrument.

The concept of responsiveness was first introduced by Guyatt et al^77^ and was used to describe the ability of an instrument measuring patient-centred outcomes to detect a clinically important change. Subsequently, Jaeschke et al^78^ suggested the term minimal clinical important difference to denote the smallest difference in score which patients perceive as being beneficial. Since then, a myriad of terms have been introduced to represent similar concepts, for example, MID,^20^ minimally important change,^79^ subjectively significant difference,^80^ and clinical important difference.^81^ Despite the many variations in terminology, it has been suggested that MID is the term that is generally used in the literature.^82^

Ascertaining the MID of PROMs provides multiple benefits.^19^ Interpreting the changes in the outcome scores remains unintuitive to both the clinicians and the researchers because statistical significant differences do not reflect the inherent value of the change in score to the patient. Thus, determining the MID allows health care professionals and researchers to interpret the significance of the changes in outcome score.^21^ Furthermore, improvement or deterioration in clinical measurements does not always align or adequately represent the changes from the patient's perspective. Therefore, the MID facilitates better understanding of a patient's self-perceived changes in oral health status and OHRQoL.^33^

There are 2 main methods used to determine the MID: anchor-based methods and distribution-based methods. Anchor-based methods use an external marker of change, that is, the anchor, to identify whether the difference in outcome score is of clinical significance.^82^^,^^83^ The anchor can be objective or subjective; however, the latter is more widely used and is often operationalised in the form of a global statement of change.^82^^,^^84^ Distribution-based methods make inferences from the data collected from the patient-reported outcome instrument whilst using the distribution of the scores to calculate the MID value.^83^ These statistical approaches most commonly include the calculation of effect size, standard error of measurement, and ratios of standard deviation.^85^ The major benefit of using distribution-based methods is that no additional data are required.^82^ However, many argue that the MID of PROMs can only truly be assessed through an understanding of the patient's subjective experience. Hence, it has been suggested that different approaches should be combined to determine MID values, with distribution-based methods providing a supporting role whilst anchor-based methods provide primary evidence.^84^^,^^86^^,^^87^

MID has been thoroughly investigated in regards to various medical conditions and treatments.^77^^,^^88^, ^89^, ^90^, ^91^, ^92^ On the other hand, its appearance in OHRQoL research has been lacking.^21^ The majority of studies have solely used distributional methods.^93^, ^94^, ^95^ One of the first studies to use an anchor-based approach reported that the MID for OHIP-14 was 5 scale points for an elderly dental population.^32^ When applied to a group of periodontal patients, the MID was around 5 scale points for the OIDP index.^96^ A recent study investigating OHRQoL after oral rehabilitative treatment reported a range of values for variants of the OHIP, including 14 scale points for the OHIP-49 and 3 scale points for the OHIP-14.^97^ Only one study has investigated the MID of OHRQoL for endodontic patients; however, only distribution-based approaches were utilised.^65^ To the best of our knowledge, there are no studies evaluating MID of OHRQoL for endodontic patients using anchor-based methods. It has been emphasised that specific MID values should be interpreted within the context of a given application, with special attention paid to the OHRQoL instrument used and the characteristics of the study group.^86^

Given that the FDI is currently working on the development of an oral health measurement tool, which incoprorates patient-centred outcomes as a measure in the assessment of oral health outcomes,^2^ research on MID may signifcantly improve the clinical usefulness of such tools.

## Conclusions

A combination of PROMs with clinical and radiographic outcome measures can result in a more comprehensive understanding of the impact of endodontic treatment and the value of different treatment modalities. The evidence supports that endodontic diseases can have a negative impact on OHRQoL, and whilst endodontic treatment has been shown to enhance patients’ OHRQoL, the extent of improvements vary. To strengthen the current evidence, well-designed large-scale clinical studies are needed to determine the effect of root canal treatment on OHRQoL in comparison with alternative modes of treatment, such as extraction and/or implants. These studies should include a baseline measurement of OHRQoL, a suitable time period of assessment, and an appropriate choice of instrument. In addition, there is a need to develop endodontic-specific OHRQoL instruments to be used in tandem with generic OHRQoL instruments in future research. Last, investigating the MID is elemental for a thorough interpretation of OHRQoL measures. Understanding the MID gives insight into both the magnitude and value of change after an intervention from the patient's perspective and hence should be a prime focus of future studies.

## Author contributions

*Conceptualisation:* Jasmine Wong, Gary Shun Pan Cheung, Prasanna Neelakantan

*Literature search and initial review*: Jasmine Wong, Prasanna Neelakantan

*Writing–first draft*: Jasmine Wong, Gary Shun Pan Cheung, Prasanna Neelakantan, Angeline Lee, Colman McGrath

*Writing–review and final version*: Jasmine Wong, Gary Shun Pan Cheung, Prasanna Neelakantan, Angeline Lee, Colman McGrath

*Funding acquisition*: Gary Shun Pan Cheung, Prasanna Neelakantan

## Conflict of interest

The authors declare that they have no conflicts of interest.
